# Assessment of C-phycocyanin effect on astrocytes-mediated neuroprotection against oxidative brain injury using 2D and 3D astrocyte tissue model

**DOI:** 10.1038/srep14418

**Published:** 2015-09-24

**Authors:** Seul Ki Min, Jun Sang Park, Lidan Luo, Yeo Seon Kwon, Hoo Cheol Lee, Hyun Jung Shim, Il-Doo Kim, Ja-Kyeong Lee, Hwa Sung Shin

**Affiliations:** 1 Department of Biological Engineering, Inha University, Incheon, 402-751, Korea; 2 Department of Anatomy, Inha University School of Medicine, Incheon, 400-712, Korea

## Abstract

Drugs are currently being developed to attenuate oxidative stress as a treatment for brain injuries. C-phycocyanin (C-Pc) is an antioxidant protein of green microalgae known to exert neuroprotective effects against oxidative brain injury. Astrocytes, which compose many portions of the brain, exert various functions to overcome oxidative stress; however, little is known about how C-Pc mediates the antioxidative effects of astrocytes. In this study, we revealed that C-Pc intranasal administration to the middle cerebral artery occlusion (MCAO) rats ensures neuroprotection of ischemic brain by reducing infarct size and improving behavioral deficits. C-Pc also enhanced viability and proliferation but attenuated apoptosis and reactive oxygen species (ROS) of oxidized astrocytes, without cytotoxicity to normal astrocytes and neurons. To elucidate how C-Pc leads astrocytes to enhance neuroprotection and repair of ischemia brain, we firstly developed 3D oxidized astrocyte model. C-Pc had astrocytes upregulate antioxidant enzymes such as SOD and catalase and neurotrophic factors BDNF and NGF, while alleviating inflammatory factors IL-6 and IL-1β and glial scar. Additionally, C-Pc improved viability of 3D oxidized neurons. In summary, C-Pc was concluded to activate oxidized astrocytes to protect and repair the ischemic brain with the combinatorial effects of improved antioxidative, neurotrophic, and anti-inflammatory mechanisms.

The brain has high potential for generation of ROS since it consumes much more oxygen than other organs and contains a high content of free-radicals susceptible to poly-unsaturated fatty acids in the neuronal membrane^1^. Although essential to metabolic activity in organisms, ROS induces cellular apoptosis and necrosis via a variety of mechanisms^2^ and participates in neurodegenerative diseases including ischemic stroke^1^. Especially, stroke is a major cause of death and disability due to short of definite treatment methods^3^. Ischemic stroke, which accounts for 85% of stroke patients, occurs primarily as a result of occlusion of small blood vessels, venous thrombosis and embolism^4^,^5^. The use of thrombolytic agents is the only method currently available for treatment to date, irrespective of its severe secondary damages to the ischemic brain. Abrupt blood reperfusion following dissolution of blood clot not only generates ROS radicals that lead to cytotoxic and inflammatory damages to tissues and cells^6^. For these reasons, inhibition of ROS generation and promotion of ROS degradation has been suggested as a target for treatment of ischemic stroke. To date, neurons have mainly been studied as targets of drug development for the treatment of ischemic stroke^6^, but astrocyte activity and recovery are also important target for ischemic stroke since astrocytes occupy the highest portion of brain tissue and help neuroprotection and repair, irrespective of their glial scar formation known to prohibit axonal regeneration^7^,^8^,^9^. Moreover, in most brain disorders, excessive ROS causes astrocytes to lose their neuroprotective function and inflict further injury^10^,^11^.

Some natural antioxidants have been investigated for their ability to prevent oxidative damage to astrocytes^10^,^12^. For example, *Spirulina*, a well-known marine cyanobacterium with neuroprotective activity^13^, contains C-Pc that exerts powerful anti-oxidative effects scavenging peroxyl, alkoxyl and hydroxyl radicals^14^,^15^,^16^. C-Pc also has anti-inflammatory, anti-microbial, neuroprotective and hepatoprotective effects^16^,^17^,^18^, which are closely associated with antioxidant effects because most pathological features are accompanied by the generation of ROS radicals^19^,^20^. Therefore, antioxidant effects together with other effects of C-Pc may be an important virtue of medicine. Although the effects of C-Pc on damaged neurons have been investigated^21^, the mechanism by which it exerts antioxidant activity through astrocytes has not yet to be elucidated. Additionally, many types of cells and astrocytes have been reported to show specific features depending on the surface topography^22^,^23^. To accurately evaluate its efficiency, C-Pc should be tested on an injury model system that mimics the *in vivo* environment to ensure the specific characteristics of cells.

In this study, we first verified whether intranasal administration of C-Pc leads to reduced infarct size and improved behavioral deficits of MCAO rat. To understand how C-Pc affects oxidized astrocytes to help the neuroprotection and repair of ischemic rats, the anti-oxidative mechanism of C-Pc through primary astrocytes occurs was elucidated by cellular, enzymatic and molecular analyses as well as based on inflammatory, and neurotrophic factors. More importantly, we first established a 3D oxidative model of astrocyte tissue and confirmed the anti-oxidative mechanism by comparison with a 2D oxidative model.

## Results

### Verification of the efficacy of intranasal administration of C-Pc for neuroprotection in the post-ischemic brain

**Figure 1:**
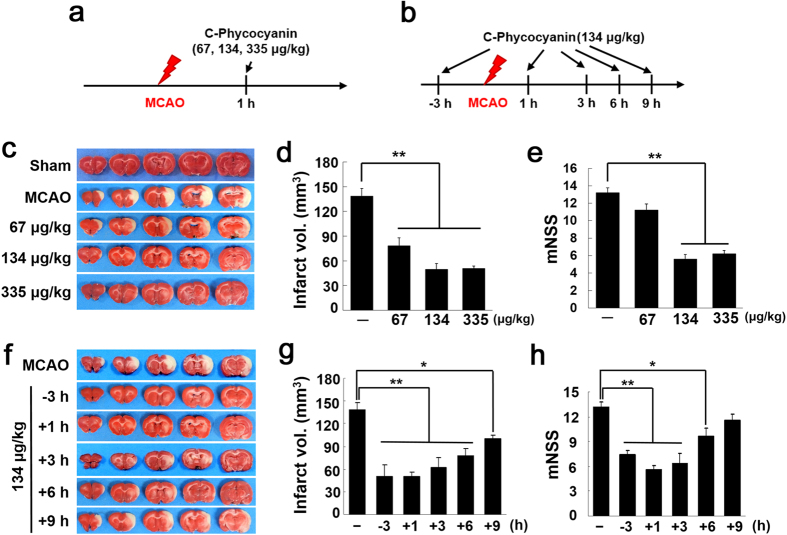
Neuroprotective effects of C-Pc in postischemic brain. (**a,b**) MCAO model was prepared and C-Pc was administered intranasally according to the presented schedules. TTC staining and neurological deficits (mNSS) evaluations were conducted 48 h post-MCAO. (**c**–**e**) C-Pc (67, 134, or 335 μg/kg) was administered intranasally at 1 h post-MCAO (n = 6–7). (**f-h**) C-Pc (134 μg/kg) was administered intranasally at 3 h prior to or 1, 3, 6 or 9 h post-MCAO (n = 5–7). (**c,f**) Representative images of infarctions in coronal brain sections are presented and (**d,g**) mean infarction volumes were assessed by TTC staining. (**e,h**) Neurological deficits were evaluated using modified neurological severity scores. Data are presented as the means ± SEMs. The asterisk denotes significant difference against MCAO sample at each concentration and time.*p < 0.05 and **p < 0.01

To investigate the neuroprotective effects of C-Pc in the post-ischemic brain, C-Pc (67, 134, or 335 μg/kg) was administered intranasally at 1 h post-MCAO and infarct volumes were assessed 2 days post-MCAO (Fig. 1a). Mean infarct volumes were reduced to 56.4 ± 7.1% (n = 6, p < 0.01), 49.5 ± 7.0% (n = 6, p < 0.01), and 50.6 ± 2.1% (n = 7, p < 0.01) of that of the untreated control (MCAO), respectively (Fig. 1c,d), demonstrating a robust neuroprotective effect of C-Pc. The administration of 134 μg/kg C-Pc at 3 h prior to or 1, 3, 6, or 9 h post-MCAO (Fig. 1b) also reduced mean infarct volumes to 36.3 ± 11.2% (n = 5, p < 0.01), 35.8 ± 5.1% (n = 6, p < 0.01), 44.7 ± 9.5% (n = 6, p < 0.01), 56.3 ± 7.0% (n = 7, p < 0.01), and 72.5 ± 3.1% (n = 5, p < 0.05) of that of the untreated control, respectively (Fig. 1f,g). Furthermore, reductions in the mean modified neurological severity score (mNSS) were observed in all C-Pc-treated animals (Fig. 1e,h). Together, these results demonstrated that C-Pc has a robust neuroprotective effect in the post-ischemic brain at all the time points containing 3 h golden time for thrombolysis in the case of human patients. Physiological parameters, namely, pH, PaO_2_, PaCO_2_, and blood glucose, were similar in C-Pc treated and untreated animal groups (Supplementary Table 2).

### Verification of safety and neuroprotective function of C-Pc in N-astrocytes

**Figure 2:**
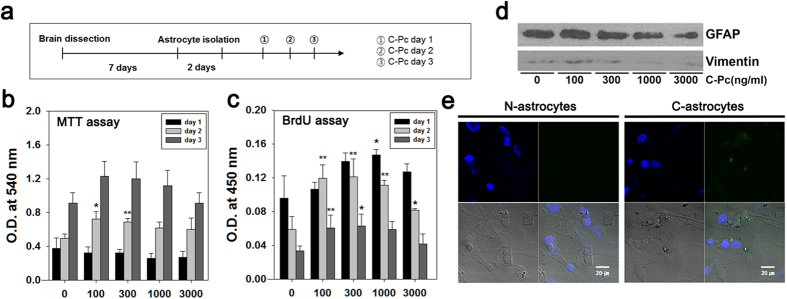
No cytotoxicity and functional improvement of C-astrocytes. (**a**) Astrocytes isolation and treatment of C-Pc were implemented according to the schedule. (**b**) Viability and (**c**) proliferation of astrocytes were assessed by MTT and BrdU assay, respectively. The concentrations of the treated C-Pc were 0, 100, 300, 1000 and 3000 ng/ml and the treatment times of C-Pc were 1, 2 and 3 days. (**d**) The quantities of GFAP and vimentin were investigated by western blot analysis at day 3 on the schedule. The full-length blots were presented in the supplementary information. (**e**) Identification of C-Pc remaining on the surface or inside the astrocytes by confocal fluorescence microscopy (800×) N-astrocytes and C-astrocytes (100 ng/ml C-Pc for 2 days). Data are presented as the means ± SEMs. The asterisk denotes significant difference against day 1 sample at each concentration. *p < 0.05 and **p < 0.01

To confirm the effects of C-Pc on normal astrocytes which are not treated with extrinsic cues (N-astrocytes), several assays were conducted according to the schedule shown in Fig. 2a. C-Pc increased the viability and proliferation of N-astrocytes, regardless of its concentration and treatment time (Fig. 2b,c). However, viability did not increase following treatment with C-Pc for 1 day since that length of time was not sufficient for the cells to divide; nevertheless, it was able to trigger self-renewal of cells via DNA synthesis. The viability and proliferation of C-Pc treated astrocytes (C-astrocytes) (100 and 300 ng/ml of C-Pc) increased obviously at day 2 relative to other concentrations of C-Pc and other days. Intermediate proteins, GFAP and vimentin were examined to evaluate the astrocytes activity when C-Pc increased the viability of N-astrocytes (Fig. 2d). Both proteins are usually analyzed to assess the status of astrocyte activation^24^. As shown in Fig. 2d, treatment with C-Pc at 100 ng/ml for 3 days induced the highest expression of GFAP and vimentin, but these values were gradually down-regulated as C-Pc concentration increased. Cultured C-astrocytes were observed by confocal microscope to confirm the adhesion region of C-Pc (Fig. 2e). Although C-Pc was found in cultured astrocytes, it did not have any adverse effects on viability and proliferation. Overall, all of these results indicate that C-Pc treatment is not toxic to astrocytes and even gives a neuroprotective effect to N-astrocytes, indicating its potential use as a clinical agent.

### Positive effects of C-Pc on the viability, proliferation, and anti-apoptosis of 2D H-astrocytes

**Figure 3:**
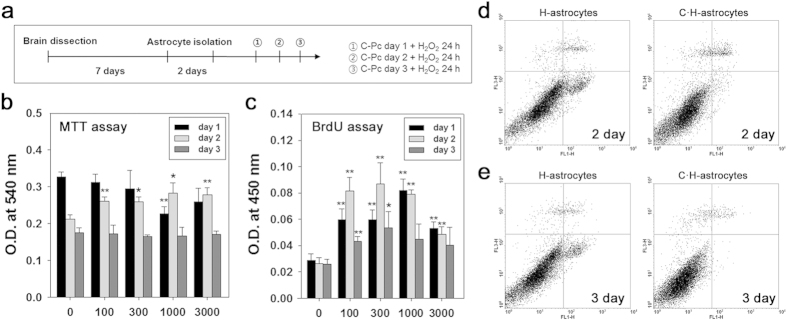
Effects of C-Pc on H-astrocytes for 24 h. (**a**) Astrocytes isolation and treatment of C-Pc and H_2_O_2_ were implemented according to the schedule. (**b**) Viability and (**c**) proliferation of astrocytes were assessed by MTT and BrdU assay, respectively. The C-Pc concentrations were 0, 100, 300, 1000, and 3000 ng/ml and the treatment times were 1, 2, and 3 day. The apoptosis rate (%) of H-astrocytes and C·H-astrocytes (300 ng/ml of C-Pc) was determined by Annexin-V and PI staining and flow cytometry at (**d**) day 2 and (**e**) day 3. Data are presented as the means ± SEMs. The asterisk denotes significant difference against day 1 sample at each concentration. *p < 0.05 and **p < 0.01

H_2_O_2_ was added during cell culture to develop *in vitro* oxidative models of astrocytes and neurons with excessive ROS. All assays were conducted according to the schedule as shown in Fig. 3a. The viability of oxidized astrocytes by H_2_O_2_ treatment (H-astrocytes) decreased, but their proliferation was not changed, and the astrocytes then overcame these conditions by treatment with C-Pc (Fig 3b,c). Viability did not decrease in response to C-Pc at 100 and 300 ng/ml on day 1, and increased following treatment with 100, 300 and 1000 ng/ml on day 2. Similarly, proliferation increased significantly on each day after treatment with C-Pc. Measurement of the rate of apoptosis by FACS revealed that H-astrocytes underwent little apoptosis at day 2 and 3 in response to 300 ng/ml of C-Pc. (Fig. 3d,e). Especially, apoptosis was greatly reduced by about 35% and 43% in response to treatment with 100 and 300 ng/ml of C-Pc, respectively, for 2 days and H_2_O_2_ for 24 h.

### Elucidation of the positive effect of C-Pc on anti-oxidative mechanism of 2D H-astrocytes

**Figure 4:**
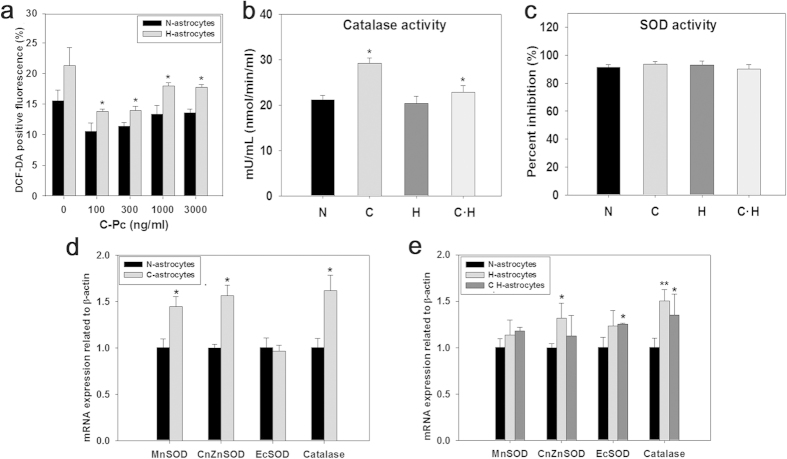
C-Pc increased anti-oxidative function via mRNA expression and activity in astrocytes under oxidative stress. (**a**) Intracellular ROS in the astrocytes treated with C-Pc for 3 days was detected by DCF-DA fluorescence levels after exposure to H_2_O_2_ for 24 h. Enzyme activities of (**b**) catalase and (**c**) total SOD were analyzed using an assay kit as described in the Online methods. (**d,e**) mRNA expression levels of MnSOD, CuZnSOD, ECSOD and catalase were evaluated by RT-qPCR. Data are presented as the means ± SEMs. The asterisk denotes significant difference against N-astrocytes sample. *p < 0.05 and **p < 0.01

ROS is modulated by the mechanisms with representative antioxidant enzymes such as superoxide dismutase (SOD) and catalase (Supplementary Fig. 2). As expected, ROS was elevated inside H-astrocytes (Fig. 4a). C- and oxidized astrocytes which were pre-treated by C-Pc (C·H-astrocytes) alleviated their ROS, leaving the lowest level at 100 ng/ml C-Pc, where C-astrocytes and C·H-astrocytes showed 35.28% and 31.92% reductions in ROS, respectively. Representative enzymes in the antioxidant pathway were analyzed by ELISA and RT-qPCR to determine which participated in the removal of ROS. As results, catalase activity of C-astrocytes and C·H-astrocytes increased compared with those of N-astrocytes and H-astrocytes, respectively (Fig. 4b). However, SOD activities of all groups of cells were not changed (Fig. 4c). C-astrocytes showed mRNA levels of MnSOD, CuZnSOD and catalase 1.44, 1.56 and 1.61 times higher than N-astrocytes, respectively (Fig. 4d). The mRNA levels of CuZnSOD and catalase in H-astrocytes were higher than in N-astrocytes but they were downregulated in C·H-astrocytes lower than C-astrocytes and H-astrocytes (Fig. 4e).

### Development of 3D H-astrocytes and Neuro2A tissue and confirmation of the positive effects of C-Pc on the anti-oxidative mechanism of astrocytes

**Figure 5:**
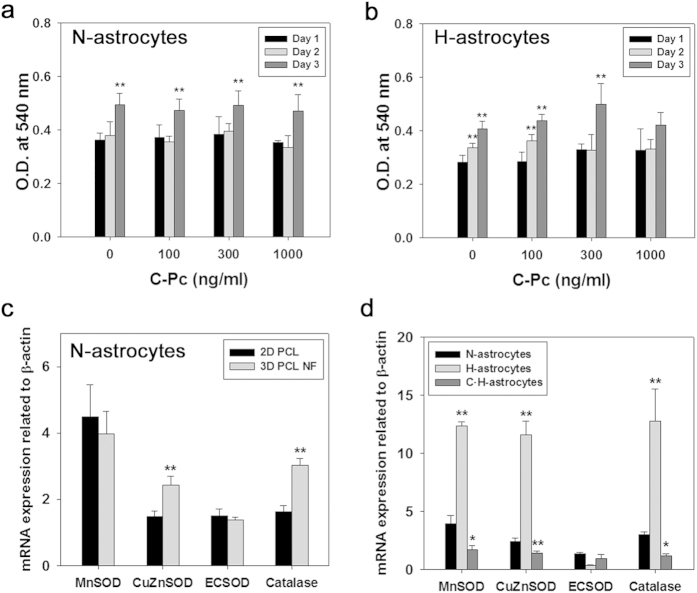
Development of *in vitro* 3D oxidative astrocytes model and confirmation of the positive effect of C-Pc on astrocytes. MTT assay was implemented for (**a**) N-astrocytes and (**b**) H-astrocytes. mRNA expression of antioxidative enzymes such as MnSOD, CuZnSOD, ECSOD and catalase on (**c**) N-astrocytes cultured on 2D PCL film and 3D PCL nanofiber and (**d**) N-astrocytes, H-astrocytes and C·H-astrocytes cultured on 3D PCL nanofiber was measured by RT-qPCR. Data are presented as the means ± SEMs. The asterisk denotes significant difference against (**a,b**) day 1 and (**d**) N-astrocytes samples at each concentration. *p < 0.05 and **p < 0.01

To provide *in vivo*-like tissue for the oxidized astrocyte model, PCL nanofiber was fabricated by electrospinning and used for cell culture, after which the samples were evaluated by MTT assay and RT-qPCR (Fig. 5). As shown in Supplementary Fig. 4, nanofiber was well fabricated for cell culture. N-astrocytes showed increased viability with time, indicating that all cells were well residing and doing their activities (Fig. 5a). H-astrocytes showed decreased viability relative to N-astrocytes, implying they were influenced by H_2_O_2_ (Fig. 5b). However, their pattern of viability increased with time, which is contrary to the gradual decrease of viability on the 2D plate. Looking over the mRNA expressions of antioxidative enzymes, 3D N-astrocytes showed higher levels of CuZnSOD and Catalase than 2D PCL N-astrocytes did, indicating that 3D nanofibrous topography enhanced the antioxidative defense system (Fig. 5c). As shown in Fig. 5d, C-astrocytes had upregulated expression of mRNA of MnSOD and ECSOD, while H-astrocytes showed upregulated MnSOD, CuZnSOD, and catalase mRNA. In general, both C-Pc and H_2_O_2_ tended to upregulate transcripts of antioxidative enzymes in both 2D and 3D culture, irrespective of the different pattern of each enzyme transcript between 2D and 3D. However, their effects were more sensitive in 3D culture. In addition C·H-astrocytes resulted in modulated mRNA levels of antioxidative enzymes below those of N-astrocytes, which is similar to the results observed for 2D culture astrocytes. For 3D cultured neurons, C-Pc also didn’t give any cytotoxic effects to the cells, looking at the non-decreased MTT results (Supplementary Fig. 1a). However, H_2_O_2_ caused a slight decrease in the viability of Neuro2A but C-Pc had the cells resist to the oxidative stress, resulting in the increase of viability (Supplementary Fig. 1b). These results shows that C-Pc could help not only astrocytes but neurons resist to oxidative stress.

### C-Pc effect on neurotrophic factors, inflammation, and glial scar of 3D H-astrocytes

**Figure 6:**
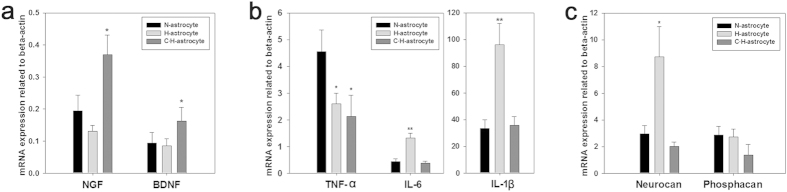
C-Pc effect on mRNA of neurotrophic factors, inflammatory factors, and glia scar of 3D H-astrocytes. The mRNA expressions of (**a**) neurotrophic factors such as BDNF and NGF, (**b**) pro-inflammatory factors such as TNF-α, IL-6 and IL-1β, and (**c**) CSPG such as Neurocan and Phosphacan were presented. Data are presented as the means ± SEMs. The asterisk denotes significant difference against N-astrocytes sample. *p < 0.05 and **p < 0.01

Neurotrophic factors, inflammation agents, and glial scar are the other cues through which astrocytes influence ischemic brain tissue. To investigate C-Pc effect on astrocytic neuroprotection through these cues, the corresponding mRNAs of astrocytes were analyzed. As results, C·H-astrocytes upregulated neurotrophic factors NGF and BDNF each 2.83 and 1.91 times those H-astrocytes, indicating C-Pc pre-treatment could contribute to recover the injury after ischemia and reperfusion (Fig. 6a). Meanwhile, the expression of IL-6 and IL-1β in H-astrocytes were up-regulated 2.96 and 2.86 times those of N-astrocytes, however C-Pc pre-treatment put back those expressions to the same levels of N-astrocytes. These imply that C-Pc suppressed inflammation of H-astrocytes. For the issue of glial scar, H-astrocytes exhibited mRNA level of neurocan 2.93 times that of N-astrocytes, while no difference was detected in phosphacan. However, both of the two mRNAs were significantly down-regulated by C-Pc pre-treatment, which indicates that the formation of glial scar was suppressed by C-Pc pre-treatment (Fig. 6c).

## Discussion

Several studies have already reported that C-Pc or *spirulina* extract reduces infarct when orally administered to the ischemia stroke animals^25^,^26^. In the present study, nasal administration of C-Pc exerted neuroprotective and repair effects on infarct brain. Nasal drug administration has recently received huge attention because it is less invasive than injection into veins and can pass the first hepatic elimination. Specifically, it can be directly administered regardless of the blood brain barrier (BBB) and is thus considered the optimum route for delivering drugs to the brain^27^,^28^. To date, the effects of intranasal administration of several drugs including EPO^29^ and tissue plasminogen activator^30^ have been demonstrated. The present study is the first to report efficacy of nasal delivery of C-Pc. Oxygen radicals make impair astrocytes, which exacerbates brain injuries such as infarct and malfunction of neuronal signals^31^. However, in order to develop a new drug, the safety of the candidate should be guaranteed essentially no matter how the efficacy such as antioxidative function appeared significant. The upregulated GFAP and vimentin indicates C-Pc activated astrocytes^32^, and which is coincident with the increased viability and proliferation and neurotrophic factors of N-astrocytes without cytotoxicity. Moreover, C-Pc also didn’t express adverse effect on neuronal viability. All these results and previous reports imply that C-Pc could be a safe candidate of drug to ischemia even though a thorough study must be still performed^25^,^33^,^34^,^35^,^36^.

To understand how C-Pc protects ischemic brain from oxidative stress, we focused on the C-Pc mediated astrocytes’ roles in neuroprotection and repair, because oxygen radicals make astrocytes impaired and which exacerbates brain injury such as infarct and malfunction of neuronal signals^31^. As seen in the 2D model of H-astrocytes, oxidant H_2_O_2_ decreased viability and proliferation and increased apoptosis (Fig. 3), similarly to the results from previous studies^37^. However, C-Pc attenuated the deterioration in viability and proliferation of H-astrocytes (Fig. 3b,c). Taken together, these results imply that C-Pc activated astrocytes to resist the harmful oxidative stress. Despite debates about the negative or positive effects of activated astrocytes in stroke^38^, C-Pc mediated activation of astrocytes was certain to exert positive effects on neuroprotection of MCAO animals (Fig. 1). Besides, astrocytes facilitate neuroprotection and repair by controlling antioxidation^39^, neurotrophic factor^40^, inflammation^41^ and glial scarring^42^. In this study, we investigated how C-Pc help astrocytes protect and repair MCAO while focusing on transcript-level studies of pre-described mechanisms.

The increased ROS after H_2_O_2_ treatment (Fig. 4a) must be implicated with the gradually reduced viability and proliferation of 2D cultured H-astrocytes (Fig. 3b,c). In response to increase of intracellular ROS, cells activate anti-oxidative mechanism as a way to defend themselves^43^. Therefore as shown in Fig. 4e, the expression of transcripts related to antioxdative enzymes in H-astrocytes more increased than N-astrocytes did. However, in spite of the defense mechanism, the activity of enzymes expected not to be continuously maintained as upregulated state since the viability was reduced but was not recovered. Indeed, the activity of SOD and catalase was not changed although only one point was measured after 24 h from H_2_O_2_ treatment (Fig. 4b,c). It could be considered that the viability of C·H-astrocytes was higher than that of H-astrocytes because C-Pc penetrated into the astrocytes (Fig. 2e) scavenged ROS, enhancing the defense system including antioxidative enzymes especially catalase activity. There was no need to upregulate mRNA of antioxidative enzymes in C·H-astrocytes since the already upregulated enzyme activity by C-Pc pre-treatment would be maintained in order to reduce the toxicity caused by H_2_O_2_.

Although the 2D results imply that C-Pc is attributed to astrocyte-mediated neuroprotection in ischemic brain, we attempted to confirm the results in 3D *in vivo*-like system. It’s because 3D nanofibrous topography helps astrocytes to express their own specific properties rather than 2D culture does^22^. As seen in Fig. 5d, the mRNA expression of antioxidative enzymes dramatically increased after treatment of H_2_O_2_. It means that the antioxidative mechanism in 3D astrocytes is more sensitive than those on 2D. This is why 3D astrocyte model was used to assess not only oxidative mechanism but also the neurotrophic factor, inflammation, and glial scar. The modulated mRNA levels of antioxidative enzymes in C·H-astrocytes was thought to be because the pre-treated C-Pc scavenged ROS or upregulated the enzyme activities at the first time but later didn’t need to maintain mRNA level high when exposed to H_2_O_2_ (Fig. 5d). Therefore, it can be concluded that C-Pc scavenges intracellular H_2_O_2_ and enhances the activity of antioxidative mechanism in both 2D and 3D system, but 3D astrocytes can be expected to validate the effectiveness of a drug candidate more explicitly because the cells reacted to oxidative environment more sensitively. Moreover, C-Pc did not show cytotoxicity to the neuronal cell line Neuro2A cultured in 3D scaffold (Supplementary Fig. 1a) and also increased the viability under oxidative stress (Supplementary Fig. 1b). These results show that C-Pc activate neurons as well as astrocytes to resist oxidative stress, leading to reduced infarct and improved behavior.

Besides to antioxidative mechanism, Additional effects of C-Pc on H-astrocytes were investigated based on 3D H-astrocyte, which is ascertained to be more sensitive to C-Pc. Astrocytes are important to the production and release of neurotrophic factors such as BDNF and NGF, which are well known to be neuroprotective and neurotrophic under neurodegenerative environments^44^,^45^. In particular, BDNF is known to exert neuroprotective effects such as anti-apoptosis, anti-inflammation, anti-neurotoxicity and neural regeneration. Therefore, C-Pc also lead astrocytes to upregulate BDNF and NGF to protect ischemic brain from oxidative stress (Fig. 6a). Glial scar has been under debate about its positive or negative roles in brain injury. Nevertheless, at chronic phase, the glial scar is generally regarded to be a barrier against neurite outgrowth^46^. In this sense that C-astrocytes and C·H-astrocytes attenuated CSPG such as Neurocan and Phosphacan expression, C-Pc would be advantageous to nerve regeneration inhibiting glial scar formation. Inflammation should be also discussed to understand C-Pc effect on ischemia because it is involved highly in the post-events of ischemia-reperfusion injury^47^. As a result, transcripts of IL-6 and IL-1β were extremely up-regulated by treatment of H_2_O_2_ but, down-regulated when pre-treated with C-Pc (Fig. 6b). According to the previous study, the infarct volume was reduced and behavior recovery was promoted compared to the control after proinflammatory factor IL-1 knockout^48^. Thus it could be considered that C-Pc in this study also regulate inflammation to influence reduction of infarct and behavior recovery.

In this study, we demonstrated the efficacy of intranasal administration of C-Pc into MCAO-rats and evaluated the effects of C-Pc on neuroprotective and repair mechanisms of H-astrocytes for the first time. Moreover, 3D astrocyte model was ascertained to be more sensitive to oxidation and thus was firstly used to assess molecular-level assessment of C-Pc effects. C-Pc was shown to enhance the viability, proliferation and activity of the H-astrocytes and the neuronal cell line, Neuro2A, treated with H_2_O_2_ without toxicity. As expected, some discrepancies between 2D and 3D were detected in transcript levels related to the antioxidative mechanism, but C-Pc was confirmed to strengthen the antioxidative mechanism of oxidized astrocytes. In addition, C-Pc led astrocytes to upregulate the transcript levels related to neurotrophic factors and to regulate inflammatory transcripts and the glial scar CSPG. In conclusion, C-Pc could be non-invasively administered (intranasally) to treat oxidative brain injuries, and was shown to strengthen local astrocytes around brain injury sites, improving resistance to the oxidative stress associated with injury.

## Online Methods

### Ethical statement

All animal studies were conducted in strict adherence with the Guide for the Care and Use of Laboratory Animals recommended by the National Institutes of Health and in compliance with the ARRIVE guidelines (http://www.nc3rs.org/ARRIVE). Animal protocols conformed to the INHA-IACUC (Inha University-Institutional Animal Care and Use Committee) with respect to ethics (Approval Number INHA 120410-137). All animal experiments were conducted on blinding.

### Preparation of MCAO rat and C-Pc nasal administration

Eight-week-old male Sprague-Dawley rats (OrientBio, Korea) were raised under diurnal lighting conditions and provided with food and tap water ad libitum. An animal model of the middle cerebral artery occlusion (MCAO) was generated as previously described^49^. Briefly, a nylon suture (4–0; AILEE, Busan, South Korea) was inserted into the right intimal carotid artery of Eight-week-old rats (250–300 g) to block MCA, after which a clip was used to occlude the common carotid for 1 h. Infarct was then induced in some parts of the right site during reperfusion. Next, intranasal administration of C-Pc (Sigma-Aldrich, USA) to the rat was performed using a pipette to inject the compound directly through the nose, after which it was transferred to the brain via the internal nasal mucosa.

### Evaluation of infarct volume by TTC staining

Brain slices were prepared by coronal sectioning with a depth of 2 mm, then stained with 1% 2, 3, 5-triphenyltetrazolium chloride (TTC) (Sigma-Aldrich, USA) at 37 °C for 15 min, after which they were fixed with 4% paraformaldehyde (Sigma-Aldrich, USA). Infarcted areas of the brain slices were analyzed using the Scion Image program (Scion Corporation, Frederick, MD, USA). To correct edema in the brain after ischemia, the analyzed infarct area was adjusted by multiplying the values of the areas of the contralateral hemisphere by the areas of the ipsilateral hemisphere. Total infarct volumes (in mm^3^) were analyzed by determining the sum of the size of each infarct containing tissue section.

### Evaluation of mNSS (modified neurological deficit severity scores)

Neurological deficits were assessed using the modified the neurological severity score (mNSS) as previously described^50^. The mNSS system consists of balance, sensory, motor and reflex tests and results are classified on a scale of 0 to 18 (normal: 0, maximum deficit: 18).

### Preparation and culture of primary astrocytes and neuronal cell line

Primary astrocytes were isolated from the brain cortex of 1-day-old Sprague-Dawley rats (SAMTACO, Korea)^51^. Briefly, the brains were homogenized in PBS using a pasteur pipette after fully removing the meninges. The homogenized brain extracts were then passed through a cell strainer with a 70 μm pore size (BD Falcon, USA), after which mixed brain cells were obtained by centrifugation at 1000 rpm for 5 min. The supernatants were subsequently removed and the cell pellets were suspended in Dulbecco’s modified eagle’s medium (DMEM) (Gibco, USA) containing 10% fetal bovine serum (FBS) (Gibco, USA) and 1% Penicillin-Streptomycin (10,000 U/ml) (Gibco, USA). Next, the cell mixture was plated on a tissue culture plate (TCPS) (SPL Life Science, Korea) for 2D culture of astrocytes and incubated at 37 °C in a 5% CO_2_ incubator for 7 days. Finally, primary astrocytes were obtained by shaking the TCPS at 200 rpm for 48 h to remove the non-attached cells (microglia and oligodendrocytes). Conditioned and fresh medium at a ratio of 2:8 were used to exchange the media. To investigate the viability and proliferation of astrocytes, cells were seeded at a density of 5 × 10^3^ cells/well and cultured in 96-well plates containing 200 μl culture media. For the ROS assay, 1 × 10^4^ cells/well were seeded on 24-well plates with 1 ml culture media. For measurement of apoptosis, western blot analysis of GFAP and vimentin and RT-qPCR, 1.25 × 10^5^ cells/well cells were seeded on 25T plates with 5 ml culture media. To investigate the effects on astrocytes, samples were incubated with 0, 100, 300, 1000, or 3000 ng/ml C-Pc for 24, 48 or 72 h. To confirm whether C-Pc pretreatment exerted protective effects against oxidative stress on astrocytes, each C-astrocytes culture was further incubated with culture media containing 200 μM H_2_O_2_ under 5% CO_2_ at 37 °C for 1 day. Neuro2A cells (mouse neuroblastroma cell line) were obtained from the American Type Culture Collection (ATCC, Manassas, VA, USA). Neuro2A was cultured with DMEM containing 10% FBS and 1% penicillin-streptomycin at 37 °C in 5% CO_2_ incubator. Neuro2A was seeded in 24-well culture plates at a density of 1 × 10^4^ cells/well and after that the media was changed to DMEM with 2% FBS, 1% PS and 20 μM retinoic acid for 5–7 days to induce differentiation. To investigate the effects of C-Pc on the differentiated Neuro2A, samples were pretreated with 100, 300 and 1000 ng/ml C-Pc for 24, 48, or 72 h before treatment with 200 μM H_2_O_2_ for 24 h.

### Fabrication of 3D PCL nanofibrous mat

A polycaprolactone (PCL) (Mn 80,000) (Sigma-Aldrich, USA) 3D nanofibrous mat and 2D PCL were fabricated as previously described^22^. Briefly, PCL 15 wt% was dissolved in a mixture of tetrahydrofuran (THF, Daejung, Korea) and *N.N*-Dimethylformamide (DMF, Daejung, Korea) at a ratio of 7:3. The PCL solution was then loaded into a syringe fixed with an 18 gauge needle and ejected at a flow rate of 1 ml/h under a 15 kV electrical field. The distance between the collector and needle tip was 15 cm. PCL solution was subsequently electrospun to oxygen plasma treated 1.9 cm^2^ glasses fixed on the collector. For 2D PCL, the nanofiber was incubated at 60 °C for 20 min. The PCL 3D nanofibrous mat fabricated on the glass was sterilized with 70% EtOH for 3 h, then washed with distilled water three times for 5 minutes and put in the 24 well plate. Finally, the nanofibrous mat was soaked in serum free medium overnight before seeding astrocytes.

### Evaluation of cell viability by MTT assay

An MTT assay was conducted to confirm the viability of C-astrocytes. Briefly, astrocytes were incubated in MTT solution diluted ten times in media (200 μl for 96 well plates and 500 μl for 24 well plates). The samples were subsequently treated with dimethyl sulfoxide (DMSO) (200 ml of and shaken 1 ml) at 100 rpm for 1 h to dissolve the purple formazan product inside the cells. Finally, the absorbance was measured at 540 nm using a Sunrise ELISA reader (Tecan, Austria).

### Evaluation of cell proliferation by BrdU assay

To evaluate cell proliferation, BrdU assays were conducted using kits according to the manufacturer’s protocols (Roche Applied Science, Indianapolis, IN, USA). Briefly, 10 μl/well of BrdU labeling solution was added to the cells, after which they were incubated at 37 °C in a 5% CO_2_ incubator for 2 h. The labeling medium was then replaced with 200 μl FixDenat solution, after which the samples were incubated for 30 min at room temperature. The FixDenat solution was then replaced with 100 μl/well of anti-BrdU-POD working solution and the samples were incubated for 90 min at room temperature. The samples were subsequently rinsed three times with 200 μl/well of PBS and reacted with 100 μl/well of substrate solution for 30 min at room temperature, after which 25 μl/well 0.1 M sulfuric acid was added to the sample and shaken at 300 rpm for 1 min to stop the reaction. To confirm the reaction, the absorbance of the sample at 450 nm was measured in an ELISA reader.

### Colocalization of fluorophores

To determine if pretreated C-Pc exists inside cells and protects H-astrocytes, samples were treated with C-Pc at 100 ng/ml and observed by confocal microscopy (LSM 510 META, Germany). Briefly, the culture media was removed from the plates and was washed gently with PBS, after which C-astrocytes were fixed with 4% paraformaldehyde for 20 min. After washing with PBS again, cells were treated with 4′-6-diamidino-2-phenylindole (DAPI) solution that made the cell nuclei visible at an excitation wavelength of 460 nm. Finally, the cytoskeleton cell morphology was viewed under a bright field microscope (Micros, Austria) and the autofluorescent C-Pc was observed at an excitation of 620 nm and emission of 650 nm.

### Analysis of cell apoptosis by annexin-V and PI staining assay (FACS)

Flow cytometry was conducted using a FACS Calibur cytometer (BD Biosciences, San Jose, CA, USA) for apoptosis analysis. Briefly, prepared cells were harvested after incubation with trypsin-EDTA (Gibco, USA) for 3 min by centrifugation at 1000 *g* for 5 min. Cells were then washed with PBS, after which they were stained for 15 min using 10% annexin V (BD pharmingen, USA) and 10% propidium iodide (PI) (Sigma-Aldrich, USA) in annexin V binding buffer (BD Biosciences, USA).

### Determination of ROS formation

Reactive oxygen species (ROS) were measured using the fluorescent reagent, CM-H_2_DCFDA (Invitrogen, Carlsbad, CA, USA), which is hydrolyzed into DCF-H and then converted into a fluorescent DCF via reaction with ROS. Briefly, the fluorescent reagent was recast to DMSO and diluted in PBS before samples containing CM-H_2_DCFDA at a final concentration of 10 μM were incubated at 37 °C in a 5% CO_2_ incubator for 15 min. Illuminated ROS were then observed by fluorescent microscope after reagent solution was replaced with PBS. To quantify the fluorescence rate, some cells were treated with fluorescent reagent and then analyzed by BCA protein assay (Sigma Aldrich, USA) to equalize the total protein levels of each sample. The cells were treated with RIPA buffer to cell induce lysis and incubated at 4 °C for 30 min. Finally, the sample was observed using a fluorescent reader at an excitation of 492 nm and an emission of 527 nm.

### Measurement of activities of antioxidative enzymes

SOD and Catalase activities were measured by each corresponding assay kit (SOD, ab65354; Catalase, ab83464) (Abcam, USA) according to the manufacturer’s recommendations. Briefly, the counted cells (2 × 10^6^) were lysed using cold lysis buffers according to each enzyme, and then incubated in order to complete the enzyme reaction. The absorbance was measured at 570 nm for catalase and at 450 nm for SOD using a microplate reader. (Tecan, Austria).

### Western blot analysis of GFAP and vimentin as specific markers for astrocytes

Samples were treated with trypsin-EDTA and lysed with RIPA lysis buffer to obtain protein, which was then quantified using a Pierce BCA Protein assay kit (Thermo Scientific, USA). Next, the proteins were electrophoresed in 12% acrylamide gel, transferred to a methanol-activated PVDF membrane and blocked with 5% skim milk in TBS at 4 °C overnight. Subsequently, primary rabbit anti-GFAP (polyclonal IgG antibodies, ab7260, Abcam) and rabbit anti-vimentin (polyclonal IgG antibodies, sc-5565, Santa Cruz) were mixed with 5% skim milk in TBS at a ratio of 1:50,000 (GFAP) and 1:500 (vimentin), respectively, then incubated at room temperature for 2 h. The membranes were subsequently washed two times with TBST for 5 min and 10 min, respectively. Next, secondary antibodies for both proteins, goat anti-(rabbit) IgG-HRP conjugated antibodies, were diluted in the 5% skim milk in TBS at a ratio of 1:5,000 (sc-2004, Santa Cruz Biotechnology, Santa Cruz, CA, USA) and applied at room temperature for 1 h. Finally, samples were reacted with ECL solution (RPN2232, GE Healthcare, UK) for 5 min, after which the bands were developed on film (Biomax Light Film, Kodak, USA).

### RT-qPCR for analysis of the expression of mRNA transcripts

mRNA transcripts were analyzed using an RNeasy Plus Mini Kit (Qiagen, Germany) to extract total RNA from astrocytes according to the manufacturer’s instructions. The remaining DNA was eliminated by incubation of the total RNA with Recombinant DNase (TAKARA, Japan) at 37 °C for 1 h. The cleaned RNA was then subjected to cDNA synthesis using an RNA QuantiFast Reverse Transcription Kit (Qiagen, Germany). Additionally, a QuantiFast SYBR Green PCR Kit (Qiagen, Germany) was used for real-time PCR assays, which were conducted on a Rotor-Gene Q PCR machine (Qiagen, Germany). Real-time PCR conditions consisted of initial denaturation at 95 °C for 5 min followed by 40 cycles of 95 °C for 10 sec and 60 °C for 30 sec.

### Statistical analysis

All experiments were performed three times independently, and the average and standard deviation were determined by Sigma Plot^TM^. Groups were compared using a student’s t-test, and a p-value < 0.05 and 0.01 was considered to indicate significance.

## Additional Information

**How to cite this article**: Min, S. K. *et al.* Assessment of C-phycocyanin effect on astrocytes-mediated neuroprotection against oxidative brain injury using 2D and 3D astrocyte tissue model. *Sci. Rep.* **5**, 14418; doi: 10.1038/srep14418 (2015).

## Supplementary Material

Supplementary Information

## References

[b1] Gorman A. M., McGowan A., O’Neill C. & Cotter T. Oxidative stress and apoptosis in neurodegeneration. J. Neurol. Sci. 139, Supplement 45–52 (1996). 8899658 10.1016/0022-510x(96)00097-4

[b2] Dröge W. Free radicals in the physiological control of cell function. Physiol. Rev. 82, 47–95 (2002). 11773609 10.1152/physrev.00018.2001

[b3] Donnan G. A., Fisher M., Macleod M. & Davis S. M. Stroke. Lancet 371, 1612–1623 (2008) 18468545 10.1016/S0140-6736(08)60694-7

[b4] de Abreu T. T., Mateus S. & Correia J. Therapy implications of transthoracic echocardiography in acute ischemic stroke patients. Stroke 36, 1565–1566 (2005). 15947277 10.1161/01.STR.0000170636.08554.49

[b5] Sacco R. L. *et al.* Guidelines for Prevention of Stroke in Patients With Ischemic Stroke or Transient Ischemic Attack A Statement for Healthcare Professionals From the American Heart Association/American Stroke Association Council on Stroke: Co-Sponsored by the Council on Cardiovascular Radiology and Intervention: The American Academy of Neurology affirms the value of this guideline. Circulation 113, e409–e449 (2006). 16534023

[b6] Nagayama T. *et al.* Cannabinoids and neuroprotection in global and focal cerebral ischemia and in neuronal cultures. J. Neurosci. 19, 2987–2995 (1999). 10191316 10.1523/JNEUROSCI.19-08-02987.1999 PMC6782289

[b7] Takano T., Oberheim N., Cotrina M. L. & Nedergaard M. Astrocytes and ischemic injury. Stroke 40, S8–S12 (2009). 19064795 10.1161/STROKEAHA.108.533166 PMC2653262

[b8] Panickar K. S. & Norenberg M. D. Astrocytes in cerebral ischemic injury: morphological and general considerations. Glia 50, 287–298 (2005). 15846806 10.1002/glia.20181

[b9] Sofroniew M. V. & Vinters H. V. Astrocytes: biology and pathology. Acta Neuropathol. 119, 7–35 (2009). 20012068 10.1007/s00401-009-0619-8 PMC2799634

[b10] Li Y. *et al.* Catalpol protects primary cultured astrocytes from *in vitro* ischemia-induced damage. Int. J. Dev. Neurosci. 26, 309–317 (2008). 18337048 10.1016/j.ijdevneu.2008.01.006

[b11] Ouyang Y.-B., Voloboueva L. A., Xu L.-J. & Giffard R. G. Selective dysfunction of hippocampal CA1 astrocytes contributes to delayed neuronal damage after transient forebrain ischemia. J. neurosci. 27, 4253–4260 (2007). 17442809 10.1523/JNEUROSCI.0211-07.2007 PMC3140959

[b12] Bi J. *et al.* Protective effects of catalpol against H_2_O_2_-induced oxidative stress in astrocytes primary cultures. Neurosci. Lett. 442, 224–227 (2008). 18652878 10.1016/j.neulet.2008.07.029

[b13] Thaakur S. & Sravanthi R. Neuroprotective effect of Spirulina in cerebral ischemia–reperfusion injury in rats. J. Neural Transm. 117, 1083–1091 (2010). 20700612 10.1007/s00702-010-0440-5

[b14] Khan M. *et al.* C-phycocyanin ameliorates doxorubicin-induced oxidative stress and apoptosis in adult rat cardiomyocytes. J. Cardiovasc. Pharm. 47, 9–20 (2006). 10.1097/01.fjc.0000191520.48404.27 16424780

[b15] Patel A., Mishra S. & Ghosh P. Antioxidant potential of C-phycocyanin isolated from cyanobacterial species Lyngbya, Phormidium and Spirulina spp. Indian J. Biochem. Biophys. 43, 25 (2006). 16955748

[b16] Romay C. *et al.* Antioxidant and anti-inflammatory properties of C-phycocyanin from blue-green algae. Inflamm. Res. 47, 36–41 (1998). 9495584 10.1007/s000110050256

[b17] Romay C., Gonzalez R., Ledon N., Remirez D. & Rimbau V. C-phycocyanin: a biliprotein with antioxidant, anti-inflammatory and neuroprotective effects. Curr. Protein Pept. Sci. 4, 207–216 (2003). 12769719 10.2174/1389203033487216

[b18] Nagaraj S. *et al.* Hepatoprotective and Antioxidative Effects of C-Phycocyanin in CCL Induced Hepatic Damage Rats. Academic J. Cancer Res. 4, 29–34 (2011).

[b19] Halliwell B. Antioxidants in human health and disease. Annu. Rev. Nutr. 16, 33–50 (1996). 8839918 10.1146/annurev.nu.16.070196.000341

[b20] Halliwell B. Free radicals and other reactive species in disease. (Wiley Online Library, 2005).

[b21] Marín-Prida J. *et al.* C-Phycocyanin protects SH-SY5Y cells from oxidative injury, rat retina from transient ischemia and rat brain mitochondria from Ca ^2+^/phosphate-induced impairment. Brain Res. Bull. 89, 159–167 (2012). 22982368 10.1016/j.brainresbull.2012.08.011

[b22] Min S. K. *et al.* Effect of topography of an electrospun nanofiber on modulation of activity of primary rat astrocytes. Neurosci. Lett. 534, 80–84 (2013). 23178191 10.1016/j.neulet.2012.11.015

[b23] Yang Y., Kulangara K., Lam R. T. S., Dharmawan R. & Leong K. W. Effects of Topographical and Mechanical Property Alterations Induced by Oxygen Plasma Modification on Stem Cell Behavior. ACS nano 6, 8591–8598 (2012). 22970773 10.1021/nn301713d

[b24] Pekny M. & Nilsson M. Astrocyte activation and reactive gliosis. Glia 50, 427–434 (2005). 15846805 10.1002/glia.20207

[b25] Pentón-Rol G. *et al.* C-Phycocyanin is neuroprotective against global cerebral ischemia/reperfusion injury in gerbils. Brain Res.Bull. 86, 42–52 (2011). 21669260 10.1016/j.brainresbull.2011.05.016

[b26] Wang Y. *et al.* Dietary supplementation with blueberries, spinach, or spirulina reduces ischemic brain damage. Exp. Neurol. 193, 75–84 (2005). 15817266 10.1016/j.expneurol.2004.12.014

[b27] Pires A., Fortuna A., Alves G. & Falcão A. Intranasal drug delivery: how, why and what for? J. Pharm. Pharm. Sci. 12, 288–311 (2009). 20067706 10.18433/j3nc79

[b28] Türker S., Onur E. & Ózer Y. Nasal route and drug delivery systems. Pharm. World Sci. 26, 137–142 (2004). 15230360 10.1023/b:phar.0000026823.82950.ff

[b29] Merelli A., Caltana L., Lazarowski A. & Brusco A. Experimental evidence of the potential use of erythropoietin by intranasal administration as a neuroprotective agent in cerebral hypoxia. Drug Metabol Drug Interact. 26, 65–69 (2011). 21756166 10.1515/DMDI.2011.007

[b30] Liu Z. *et al.* Subacute intranasal administration of tissue plasminogen activator increases functional recovery and axonal remodeling after stroke in rats. Neurobiol. Dis. 45, 804–809 (2012). 22115941 10.1016/j.nbd.2011.11.004 PMC3259280

[b31] Chen Y. & Swanson R. A. Astrocytes and brain injury. J. Cerebr. Blood F. Met. 23, 137–149 (2003). 10.1097/01.WCB.0000044631.80210.3C 12571445

[b32] East E., Golding J. P. & Phillips J. B. A versatile 3D culture model facilitates monitoring of astrocytes undergoing reactive gliosis. J. Tissue Eng. Regen. Med. 3, 634–646 (2009). 19813215 10.1002/term.209 PMC2842570

[b33] Chen J.-C. *et al.* Spirulina and C-phycocyanin reduce cytotoxicity and inflammation-related genes expression of microglial cells. Nutr. Neurosci. 15, 252–256 (2012). 22687570 10.1179/1476830512Y.0000000020

[b34] Rimbau V. *et al.* C-phycocyanin protects cerebellar granule cells from low potassium/serum deprivation-induced apoptosis. N-S. Arch. Pharmacol. 364, 96–104 (2001). 10.1007/s002100100437 11534860

[b35] Rimbau V., Camins A., Romay C., González R. & Pallàs M. Protective effects of C-phycocyanin against kainic acid-induced neuronal damage in rat hippocampus. Neurosci. Lett. 276, 75–78 (1999). 10624795 10.1016/s0304-3940(99)00792-2

[b36] Guo Y.-L., Ding X.-J., Sun F. & WANG C. Neuroprotective effects and mechanism of phycocyanin in cerebral ischemia reperfusion injury in rats. Chinese J. Marine Drugs 25, 20 (2006).

[b37] Bretón R. R. & Rodríguez J. C. G. Excitotoxicity and oxidative stress in acute ischemic stroke, Acute Ischemic Stroke. (Prof. Julio Cesar Garcia Rodriguez Ed.) (2012).

[b38] Barreto G., White R. E., Ouyang Y., Xu L. & Giffard R. G. Astrocytes: targets for neuroprotection in stroke. Cent. Nerv. Syst. Agents Med. Chem. 11, 164 (2011). 21521168 10.2174/187152411796011303 PMC3167939

[b39] Wilson J. X. Antioxidant defense of the brain: a role for astrocytes. Can. J. Physiol. Pharm. 75, 1149–1163 (1997). 9431439

[b40] Saha R. N., Liu X. & Pahan K. Up-regulation of BDNF in astrocytes by TNF-α: a case for the neuroprotective role of cytokine. J. Neuroimmune Pharmacol. 1, 212–222 (2006). 18040799 10.1007/s11481-006-9020-8 PMC2131740

[b41] Allan S. M. & Rothwell N. J. Inflammation in central nervous system injury. Philos. T. Roy. Soc. B 358, 1669–1677 (2003). 10.1098/rstb.2003.1358 PMC1693261 14561325

[b42] Rolls A., Shechter R. & Schwartz M. The bright side of the glial scar in CNS repair. Nat. Rev. Neurosci. 10, 235–241 (2009). 19229242 10.1038/nrn2591

[b43] Park J. S. *et al.* Antioxidant mechanism of isoflavone metabolites in hydrogen peroxide‐stimulated rat primary astrocytes: critical role of hemeoxygenase‐1 and NQO1 expression. J. Neurochem. 119, 909–919 (2011). 21781119 10.1111/j.1471-4159.2011.07395.x

[b44] Hefti F., Hartikka J. & Knusel B. Function of neurotrophic factors in the adult and aging brain and their possible use in the treatment of neurodegenerative diseases. Neurobiol. Aging 10, 515–533, (1989). 2682327 10.1016/0197-4580(89)90118-8

[b45] Chen A., Xiong L.-J., Tong Y. & Mao M. The neuroprotective roles of BDNF in hypoxic ischemic brain injury (Review). Bio. Rep. 1, 167–176 (2013). 10.3892/br.2012.48 PMC3956206 24648914

[b46] Jurynec M. J. *et al.* TIGR is upregulated in the chronic glial scar in response to central nervous system injury and inhibits neurite outgrowth. Mol. Cell. Neurosci. 23, 69–80 (2003). 12799138 10.1016/s1044-7431(03)00019-8

[b47] McCord J. Oxygen-derived radicals: a link between reperfusion injury and inflammation. Faseb. J. 46, 2402–2406 (1987). 3032690

[b48] Boutin H. *et al.* Role of IL-1α and IL-1β in ischemic brain damage. J. Neurosci. 21, 5528–5534 (2001). 11466424 10.1523/JNEUROSCI.21-15-05528.2001 PMC6762680

[b49] Bederson J. B. *et al.* Rat middle cerebral artery occlusion: evaluation of the model and development of a neurologic examination. Stroke 17, 472–476 (1986). 3715945 10.1161/01.str.17.3.472

[b50] Chen J. *et al.* Intravenous administration of human umbilical cord blood reduces behavioral deficits after stroke in rats. Stroke 32, 2682–2688 (2001). 11692034 10.1161/hs1101.098367

[b51] Fedoroff S. & Richardson A. Protocols for neural cell culture. (Springer, 2001).

