# Supplementary material for: Assessment of C-phycocyanin effect on astrocytes-mediated neuroprotection against oxidative brain injury using 2D and 3D astrocyte tissue model
Source: Sci Rep. 2015 Sep 24;5:14418. doi: 10.1038/srep14418 (PMC4585836; doi:10.1038/srep14418)
Supplement: Supplementary Information [file srep14418-s1.pdf]

Assessment of C-phycocyanin effect on astrocytes-mediated neuroprotection against oxidative brain injury using 2D and 3D astrocyte tissue model

Supplementary Information

Seul Ki Min[1], Jun Sang Park[1], Lidan Luo[2], Yeo Seon Kwon[1], Hoo Cheol Lee[1], Hyun Jung Shim[1], Il-Doo Kim[2], Ja-Kyeong Lee[2], Hwa Sung Shin[1,*]

[1] Department of Biological Engineering, Inha University, Incheon, 402-751, Korea.

[2] Department of Anatomy, Inha University School of Medicine, Incheon, 400-712, Korea.

*** Corresponding author**

Hwa Sung Shin, hsshin@inha.ac.kr, Tel: +82-32-860-9221, Fax: +82-32-872-4046

This file includes :

2 tables and 4 figures which are supporting our research.

Gene	Forward (5'→3')	Reverse (5'→3')
**MnSOD**	CGGGTTTCTGAGTGAGGTCAG	GTCAGGGAAAGGGTGTCCTTC
**CuZnSOD**	TCACTTCGAGCAGAAGGCAA	TGAGGTCCTGCAGTGGTACA
**EcSOD**	GAGAGCTTGTCAGGTGTGGAA	CGGACTCTCCGGTATCTGAC
**Catalase**	GGCACAAGCCTCACCAGTAA	CCCTCGGGAAATGCCATCAA
**BDNF**	GACTCTGGAGAGCGTGAA	CCACTCGCTAATACTGTCAC
**NGF**	CTGGACTAAACTTCAGCATTC	TGTTGTTAATGTTCACCTCGC
**TNF-α**	GCTTGGTGGTTTGCTACGAC	ATGGGCTCCCTCTCATCAGT
**IL-6**	CCTATTGAAAATCTGCTCTG	TTGGGGTAGGAAGGACTATT
**IL-1β**	AAATGCCTCGTGCTGTCTGA	AGGCCACAGGGATTTTGTCG
**Neurocan**	TTTCAGTCCACAGCGATCAG	AGGAGAGGGATACAGCAGCA
**Phosphacan**	TTGACAAGTGATGAAGAGAGTGG	AATCAGCACATCTCGTTCTATCC
**β-actin**	CCGCGAGTACAACCTTCTTG	CATGCCGGAGCCGTTGTC

Table S1. mRNA sequences that were designed for the analysis of neuroprotective function of C-Pc treated astrocytes.

	Control group		MCAO group	
	Base	C-Pc treatment	Base	C-Pc treatment
Temperature, ℃	37.1 ± 0.2	37.1 ± 0.1	37.0 ± 0.1	37.1 ± 0.1
pH	7.7 ± 0.1	7.6 ± 0.1	7.6 ± 0.1	7.6 ± 0.1
pO_2, mmHg	146 ± 19.2	149.5 ± 7.3	150.2 ± 6.7	154.3 ± 13.0
pCO_2, mmHg	40.3 ± 1.5	41.3 ± 1.4	39.1 ± 1.2	38.8 ± 0.6
Glucose, mg/dL	109.8 ± 5.6	109.6 ± 2.2	109.6 ± 5.2	118.4 ± 2.4

Values are means SD (n=3). One way analysis of variance revealed no significant intergroup difference for any variance.

Table S2. Physiological parameters

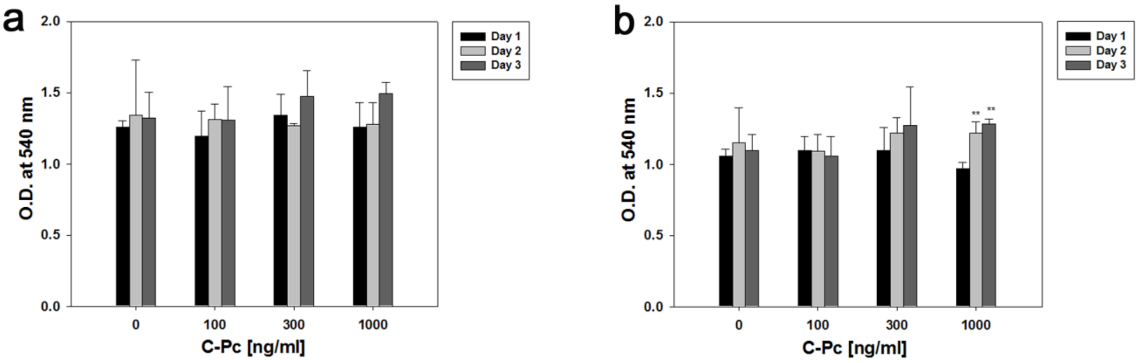

Figure S1. Effects of C-Pc on normal and oxidized Neuro2A. The C-Pc concentrations were 0, 100, 300, 1000, and 3000 ng/ml and the treatment times were 1, 2, and 3 day. (a) The viability of Neuro2A under normal condition was not changed with time and concentration of C-Pc. (b) The viability of Neuro2A under oxidative stress was also maintained and even upregulated by treatment of C-Pc. Data are presented as the means±SEMs. * $p < 0.05$ and ** $p < 0.01$ versus “day 1” at each concentration.

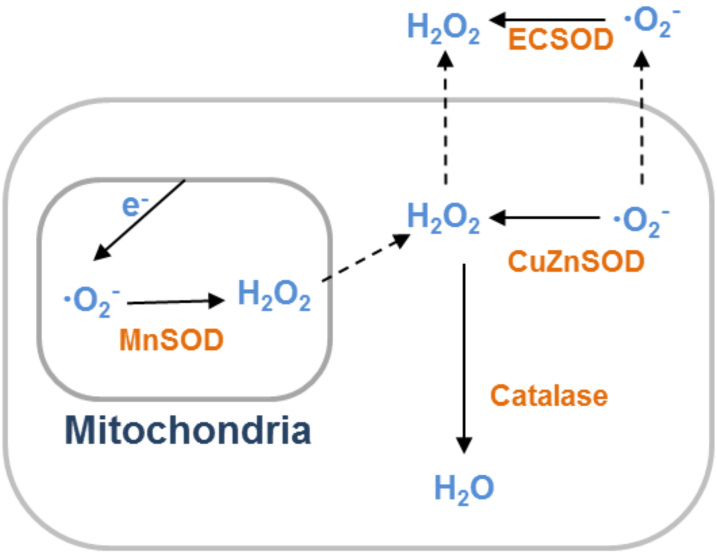

Figure S2. Typical antioxidant enzymes (MnSOD, CuZnSOD, EcSOD and Catalase) for removing ROS from the cells are marked in the metabolic pathway.

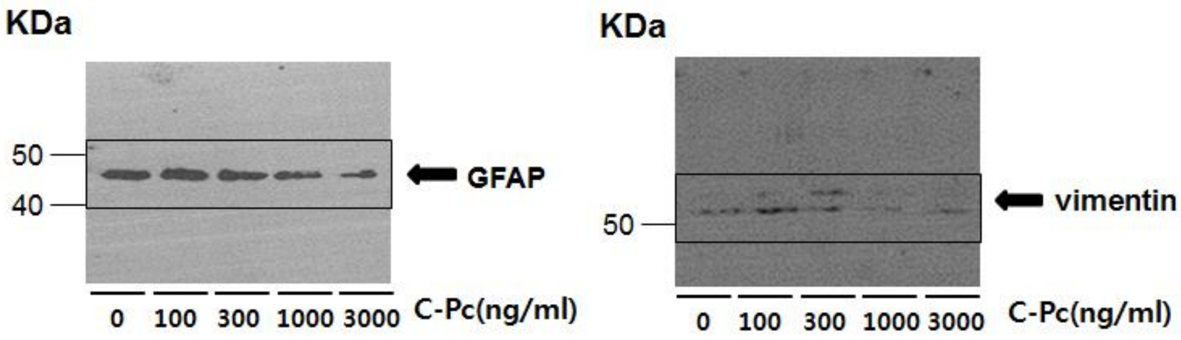

Figure S3. Full length blots of Fig. 4c

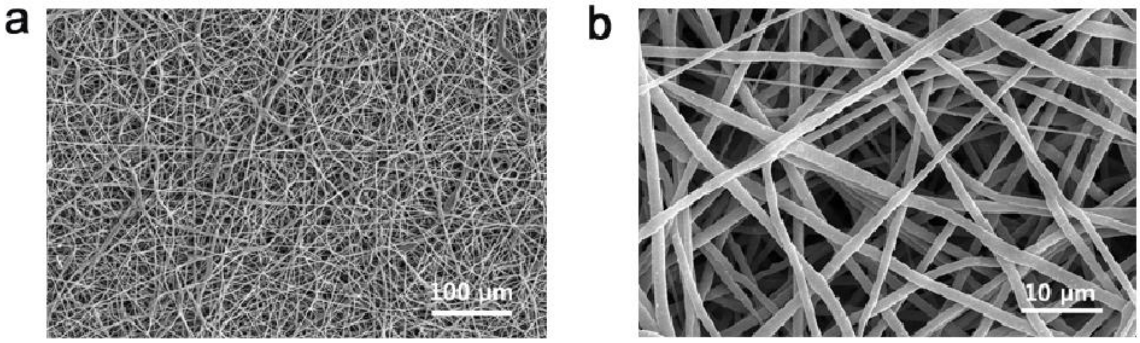

Figure S4. Fabricated electrospun nanofiber used for cell culture surface
